# Association between fluid intake and extubation failure in intensive care unit patients with negative fluid balance: a retrospective observational study

**DOI:** 10.1186/s12871-022-01708-3

**Published:** 2022-06-01

**Authors:** Tong Li, Dawei Zhou, Dong Zhao, Qing Lin, Dija Wang, Chao Wang

**Affiliations:** Department of Critical Care Medicine, Beijing Tongren Hospital, Capital Medical University, Beijing, China

**Keywords:** Extubation failure, Negative fluid balance, Fluid intake, Chronic obstructive pulmonary disease, Diuretics, Intensive care

## Abstract

**Background:**

Negative fluid balance (NFB) is associated with reduced extubation failure. However, whether achieving more NFB can further improve extubation outcome has not been investigated. This study aimed to investigate whether more NFB and restricted fluid intake were associated with extubation success.

**Methods:**

We performed a retrospective study of adult patients with mechanical ventilation (MV) admitted to Medical Information Mart for Intensive Care (MIMIC-III) from 2001 to 2012. Patients with duration of MV over 24 hours and NFB within 24 hours before extubation were included for analysis. The primary outcome was extubation failure, defined as reintubation within 72 hours after extubation. Association between fluid balance or fluid intake and extubation outcome were investigated with multivariable logistic models.

**Results:**

A total of 3433 extubation events were recorded. 1803 with NFB were included for the final analysis, of which 201(11.1%) were extubation failure. Compared with slight NFB (− 20 to 0 ml/kg), more NFB were not associated improved extubation outcome. Compared with moderate fluid intake (30 to 60 ml/kg), lower (< 30 ml/kg, OR 0.75, 95% CI [0.54, 1.05], *p* = 0.088) or higher (> 60 ml/kg, OR 1.63, 95% CI [0.73, 3.35], *p* = 0.206) fluid intake was not associated with extubation outcome. Duration of MV, chronic obstructive pulmonary disease (COPD), hypercapnia, use of diuretics, and SAPSIIscore were associated with extubation failure.

**Conclusions:**

More NFB or restricted fluid intake were not associated with reduced extubation failure in patients with NFB. However, for COPD patients, restricted fluid intake was associated with extubation success.

**Supplementary Information:**

The online version contains supplementary material available at 10.1186/s12871-022-01708-3.

## Introduction

Extubation is one of the critical procedures during the intensive care unit (ICU) stay in patients surviving from mechanical ventilation (MV) [1]. Extubation failure, defined as a need for reintubation within certain times after planned extubation, occurs in 10 to 20% of patients and is associated with extremely poor outcomes [2–5]. However, the pathophysiology remains uncertain [6].

Fluid balance, which has significant impact on clinical outcomes in several critically ill populations, has been found to be associated with extubation outcome [7–9]. Compared with positive fluid balance (PFB) before extubation, negative fluid balance (NFB) is associated with improved extubation outcomes [10–12]. However, most of the current studies focused on the comparison between PFB and NFB; whether achieving more NFB could further improve extubation outcomes has not been investigated. Furthermore, since conservative strategy of fluid management could improve lung function and shorten the duration of mechanical ventilation [13–15]; whether restricted fluid management is associated with extubation success in patients with NFB seems significantly important for weaning and extubation processes.

Based on the current evidence, the aims of the present study were to investigate whether more NFB and restricted fluid intake are associated with reduced extubation failure in mechanically ventilated patients planned for extubation with NFB.

## Methods

### Setting

This study used data stored in the high-resolution database, the Medical Information Mart for Intensive Care (MIMIC-III) database (mimic.physionet.org), which comprises 61,532 ICU admissions to Beth Israel Deaconess Medical Center from 2001 to 2012. The elaborate description of MIMIC-III is available elsewhere [16]. The MIMIC-III was exempt from institutional review board (IRB) approval due to the retrospective design, lack of direct patient intervention, and the security schema. After completing a National Institutes of Health (NIH) web-based training course, the author (certification number: 28795067) was approved to access to the database for research aims.

### Study population

All patients in the MIMIC-III database were eligible for inclusion. As for those who admitted to ICU for more than once, only the first stay was taken into consideration. We included adult patients with the first mechanical ventilation in the ICU. The exclusion criteria were: (1) Duration of mechanical ventilation < 24 hours, (2) Using the continuous renal replacement treatment (CRRT) due to the influence of calculating fluid balance, (3) Tracheotomy before or within the three days after extubation, (4) Self-extubation, (5) Missing data of fluid balance.

### Clinical variables and outcomes

Demographics (age, gender, ethnicity (White, Asian, Black, Hispanic/Latino, Other), weight), comorbidities (diabetes mellitus, hypertension, chronic heart failure, chronic obstructive pulmonary disease (COPD), liver disease, renal insufficiency, and tumor), and ICU types were extracted from the database. Furthermore, sequential organ failure assessment (SOFA) score, and simplified acute physiology score II (SAPSII) were calculated for each patient. We used the MIMIC Code Repository to define many concepts in MIMIC-III database [17]. Rapid shallow breathing index was defined as respiratory rate divided by tidal volume. Diuretics use and blood products transfusion were defined as any diuretics or blood products used within 24 hours before extubation. Duration of mechanical ventilation was defined as the difference between time of extubation and start time of mechanical ventilation.

Fluid intake and fluid output were assessed during the 24-hour period prior to extubation. Fluid balance (FB) was calculated as total fluid intake minus total fluid output. Negative fluid balance (NFB) was defined as FB < 0. Fluid intake and FB were categorized into three levels, respectively. 30 ml/kg/24 hours was used as one interval for fluid intake: level 1 (< 30 ml/kg/24 hours), level 2 (30 to 60 ml/kg/24 hours), and level 3 (> 60 ml/kg/24 hours); FB was categorized using 20 ml/kg/24 hours as one interval: level 1 (− 20 to 0 ml/kg/24 hours), level 2 (− 40 to − 20 ml/kg/24 hours), and level 3 (< − 40 ml/ kg/24 hours). Subgroup analyses were performed in patients with COPD or not and using diuretics or not.

The primary endpoint was extubation failure, defined as reintubation within 72 hours after extubation. The other study endpoints included hospital mortality, ICU length of stay, and hospital length of stay.

### Statistical analysis

Continuous variables were shown as mean and standard deviation (SD) or median and interquartile range (IQR), compared using Student’s t test or Wilcoxon rank-sum test as appropriate. Categorical variables were reported as numbers and percentages, and were analyzed with Chi-square test or Fisher’s exact test as appropriate. As for missing data, missing rate were recorded and patients with missing variable were considered as a unique group.

First, a descriptive analysis was performed between extubation success and extubation failure patients both in the overall patients and the patients with NFB. Second, differences among three fluid intake levels were analyzed. Third, multivariate logistic regression models were built with variables of a *p* value < 0.20 identified by the univariate analysis or those were considered clinically important; Next, a stepwise backward elimination method was used to remove variables with *p* value > 0.1. Potential multicollinearity was tested using the variance inflation factor and goodness of fit was tested for all logistic regression models. Fourth, subgroup analyses were performed within patients with COPD or not and diuretics use or not with multivariable logistic regression models.

A two-sided *P* value of less than 0.05 was considered statistically significant. Data extraction was performed using PostgreSQL (version 10.5) and all statistical analyses were performed using the R software (version 3.6.1, www.r-project.org).

## Results

**Fig. 1:**
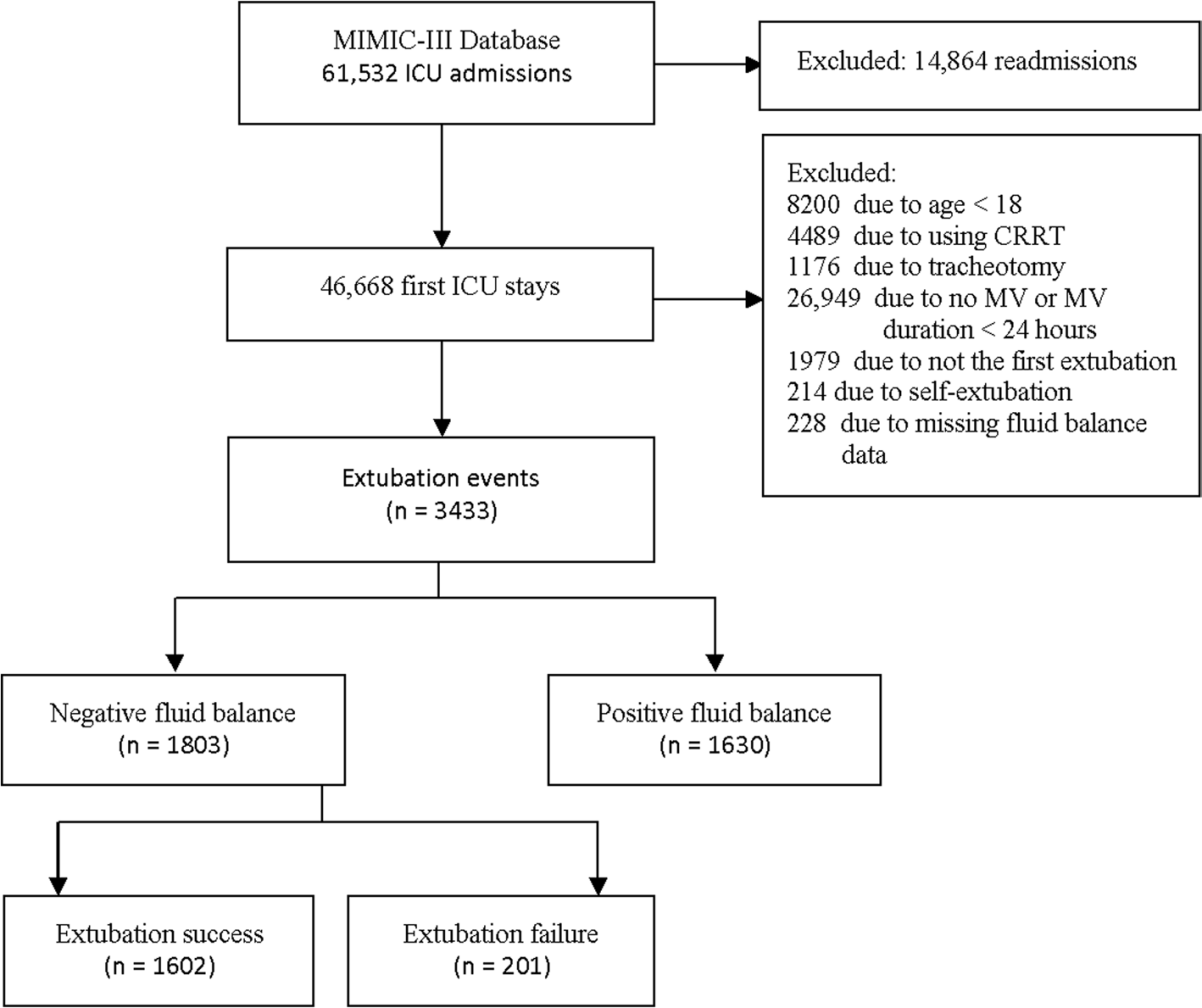
Flowchart of subject selection. MIMIC medical information mart for intensive care, CRRT continuous renal replacement therapy, ECMO extracorporeal membrane oxygenation, MV mechanical ventilation

After exclusion, a total of 3433 patients with extubation events were included for analysis, of which 1803 (52.5%) had NFB within 24 hours before extubation (Fig. 1). Compared with PFB, patients with NFB had lower rate of extubation failure (14% vs 11%, *p* = 0.028) (S-Table 1). In patients with NFB, the median age was 66 (IQR, 53–77) and 988 (55%) were male, and 201 (11.1%) patients had extubation failure. Extubation failure patients had higher percentage of COPD (33% vs 24, *p* = 0.01), higher RSBI (64 VS 59, *p* = 0.005), higher SAPS II score on the day of extubation (44 vs 41, *p* = 0.01), and higher percentage of diuretics use (51% vs 37%, *p* < 0.001). Compared with patients of extubation success, extubation failure patients also had longer duration of mechanical ventilation before extubation (106 vs 85 hours, *p* = 0.002), longer ICU and hospital length of stay, and higher hospital mortality (19% vs 11%, *p* = 0.001) (Table 1).

**Table 1:** Characteristics between extubation success and failure in patients with negative fluid balance

Variables	Total (*n* = 1803)	Extubation success (*n* = 1602)	Extubation failure (*n* = 201)	*P* value
Age, years (median, [IQR])	66 (53, 77)	66 (53, 77)	69 (57, 78)	0.055
Sex: male (n (%))	988 (55)	879 (55)	109 (54)	0.923
Weight, Kg (median, [IQR])	80 (67, 95)	80 (67, 95)	78 (66, 92)	0.353
Ethnicity, (n (%))				0.897
White	1310 (73)	1160 (72)	150 (75)	
Asian	34 (2)	32 (2)	2 (1)	
Black	146 (8)	132 (8)	14 (7)	
Hispanic/Latino	54 (3)	49 (3)	5 (2)	
Other	259 (14)	229 (14)	30 (15)	
ICU types, (n (%))				0.422
CCU	250 (14)	230 (14)	20 (10)	
CSRU	330 (18)	292 (18)	38 (19)	
MICU	693 (38)	613 (38)	80 (40)	
SICU	265 (15)	230 (14)	35 (17)	
TSICU	265 (15)	237 (15)	28 (14)	
Comorbidities, n (%)				
Diabetes Mellitus	494 (27)	433 (27)	61 (30)	0.362
Chronic Heart failure	699 (39)	619 (39)	80 (40)	0.809
Hypertension	920 (51)	815 (51)	105 (52)	0.772
COPD	453 (25)	387 (24)	66 (33)	0.01
Liver disease	165 (9)	148 (9)	17 (8)	0.816
Tumor	141 (8)	130 (8)	11 (5)	0.24
Renal insufficiency	268 (15)	237 (15)	31 (15)	0.896
PaCO_2_ before extubation				< 0.001
< 35 mmHg	248 (14)	224 (14)	24 (12)	
35–45 mmHg	860 (48)	776 (48)	84 (42)	
> 45 mmHg	480 (27)	399 (25)	81 (40)	
Missing data	215 (12)	203 (13)	12 (6)	
RSBI, breaths/min/L	59 (46, 76)	59 (45, 75)	64 (50, 81)	0.005
Disease severity score before extubation (median, [IQR])				
SOFA	6 (4, 8)	6 (4, 8)	6 (4, 8)	0.315
SAPS II	42 (33, 51)	41 (32, 51)	44 (37, 52)	0.01
Fluid balance (ml/kg/24 hours)	−15 (−27, −8)	−15 (− 27, − 8)	-15 (−24, − 7)	0.412
Total Input (ml/kg/24 hours)	24 (15, 34)	24 (15, 34)	26 (16, 36)	0.048
Total Output (ml/kg/24 hours)	41 (30, 56)	41 (30, 56)	43 (31, 56)	0.386
Diuretics, (n (%))	697 (39)	595 (37)	102 (51)	< 0.001
Blood products transfusion, (n (%))	441 (24)	381 (24)	60 (30)	0.072
MV duration before extubation, hours	87 (51, 147)	85 (50, 145)	106 (62, 156)	0.002
Hospital LOS, days (median, [IQR])	15 (9, 24)	14 (9, 22)	23 (16, 33)	< 0.001
ICU LOS, days (median, [IQR])	8 (4, 13)	7 (4, 12)	16 (11, 25)	< 0.001
Hospital mortality, n (%)	213 (12)	174 (11)	39 (19)	0.001

*IQR* interquartile range, *ICU* intensive care unit, *CCU* Coronary Care Unit, *CSRU* Cardiac Surgery Recovery Unit, *TSICU* Trauma Surgical ICU, *MICU* Medical ICU, *SICU* Surgical ICU, *COPD* chronic obstructive pulmonary disease, *RSBI* rapid shallow breathing index, *SOFA* sequential organ failure assessment score, *SAPS II* simplified acute physiology score, *LOS* length of stay

Extubation failure patients had slightly higher total input (26 vs 24 ml/kg/24 hours, *p* = 0.048) than extubation success patients; However, the two groups had similar fluid balance. For patients with three levels of fluid intake, the percentage of extubation failure and hospital mortality were similar. However, higher fluid intake was associated with longer duration of mechanical ventilation before extubation, longer ICU and hospital length of stay and lower percentage of diuretics use (Table 2).

**Table 2:** Characteristics among fluid intake categories before extubation

	Fluid intake categories before extubation			
Variables	< 30 ml/kg (*n* = 1211)	30–60 ml/kg (*n* = 531)	> 60 ml/kg (*n* = 61)	*P* value
RSBI, breaths/min/L	60 (46, 76)	58 (45, 75)	53 (42, 76)	0.393
Disease severity score before extubation (median, [IQR])				
SOFA	6 (4, 8)	5 (3, 8)	5 (3, 9)	0.009
SAPS II	42 (34, 52)	39 (30, 50)	42 (35, 55)	< 0.001
Fluid balance (ml/kg/24 hours)	−16 (−27, −8)	−14 (−27, −6)	−18 (−27, −8)	0.217
Total Input (ml/kg/24 hours)	18 (13, 24)	38 (34, 45)	67 (64, 74)	< 0.001
Total Output (ml/kg/24 hours)	35 (26, 45)	55 (45, 69)	85 (78, 99)	< 0.001
Diuretics, (n (%))	512 (42)	174 (33)	11 (18)	< 0.001
Blood products transfusion, (n (%))	288 (24)	137 (26)	16 (26)	0.631
MV duration before extubation, hours	84 (50, 137)	94 (52, 169)	98 (48, 136)	0.008
Extubation failure, (n (%))	127 (10)	64 (12)	10 (16)	0.264
Hospital LOS, days (median, [IQR])	15 (10, 23)	18 (11.5, 28)	22 (14, 37)	< 0.001
ICU LOS, days (median, [IQR])	8 (5, 14)	10 (6, 16)	10 (6, 13)	0.001
Hospital mortality, n (%)	139 (11)	64 (12)	10 (16)	0.5

*IQR* interquartile range, *ICU* intensive care unit, *RSBI* rapid shallow breathing index, *SOFA* sequential organ failure assessment score, *SAPS II* simplified acute physiology score, *LOS* length of stay

For multivariable logistic regression models, after adjusting for confounders, compared with slightly lower FB (− 20 to 0 ml/kg), more negative FB were not associated with extubation failure. Compared with moderate fluid intake (30 to 60 ml/kg), lower (OR 0.75, 95% CI [0.54, 1.05], *p* = 0.088) or higher (OR 1.63, 95% CI [0.73, 3.35], *p* = 0.206) fluid intake was not associated with extubaiton failure. Comorbidity of COPD and diuretics use were associated with extubation failure. Longer duration of mechanical ventilation had higher odds ratio for extubation failure. Higher RSBI had a trend of higher extubation failure, but was not statistically significant. Compared with normal PaCO_2_, high PaCO_2_ (> 45 mmHg) was associated with extubation failure (Table 3).

**Table 3:** Adjusted ORs for extubation failure using FB and fluid intake as design variables

Model 1 (Negative FB)			Model 2 (Fluid intake)		
Variables	OR [95% CI]	*P* value	Variables	OR [95% CI]	*P* value
Negative FB categories			Fluid intake categories		
< −40 ml/kg	1.07 [0.62, 1.75]	0.806	< 30 ml/kg	0.75 [0.54, 1.05]	0.088
-40 to −20 ml/kg	0.87 [0.61, 1.22]	0.431	30 to 60 ml/kg	Reference	
-20 to 0 ml/kg	Reference		> 60 ml/kg	1.63 [0.73, 3.35]	0.206
MV duration (per 24 hours increase)	1.05 [1.01, 1.09]	0.016	MV duration (per 24 hours increase)	1.05 [1.01, 1.09]	0.022
COPD (Yes vs No)	1.43 [1.03, 1.97]	0.031	COPD (Yes vs No)	1.48 [1.06, 2.04]	0.019
RSBI (per 10 units increase)	1.06 [1.00, 1.13]	0.052	RSBI (per 10 units increase)	1.06 [1.00, 1.13]	0.055
PaCO_2_ before extubation			PaCO_2_ before extubation		
< 35 mmHg	0.99 [0.60, 1.58]	0.962	< 35 mmHg	0.99 [0.60, 1.58]	0.959
35–45 mmHg	Reference		35–45 mmHg	Reference	
> 45 mmHg	1.66 [1.17, 2.35]	0.004	> 45 mmHg	1.73 [1.22, 2.45]	0.002
Missing	0.54 [0.28, 1.00]	0.057	Missing	0.56 [0.29, 1.02]	0.073
SAPS II	1.01 [1.00, 1.02]	0.046	SAPS II	1.01 [1.00, 1.02]	0.043
Diuretics (Yes vs No)	1.59 [1.17, 2.15]	0.003	Diuretics (Yes vs No)	1.64 [1.21, 2.23]	0.001

*OR* odds ratio, *FB* fluid balance, *MV* mechanical ventilation, *COPD* chronic obstructive pulmonary disease, *RSBI* rapid shallow breathing index, *SAPS II* simplified acute physiology score

Model 1 evaluated the association between negative fluid balance and extubation failure, with MV duration, COPD, RSBI, PaCO_2_, SAPS II, and diuretics use as confounders. Model 2 evaluated the association between fluid intake and extubation failure, with MV duration, COPD, RSBI, PaCO_2_, SAPS II, and diuretics use as confounders

**Fig. 2:**
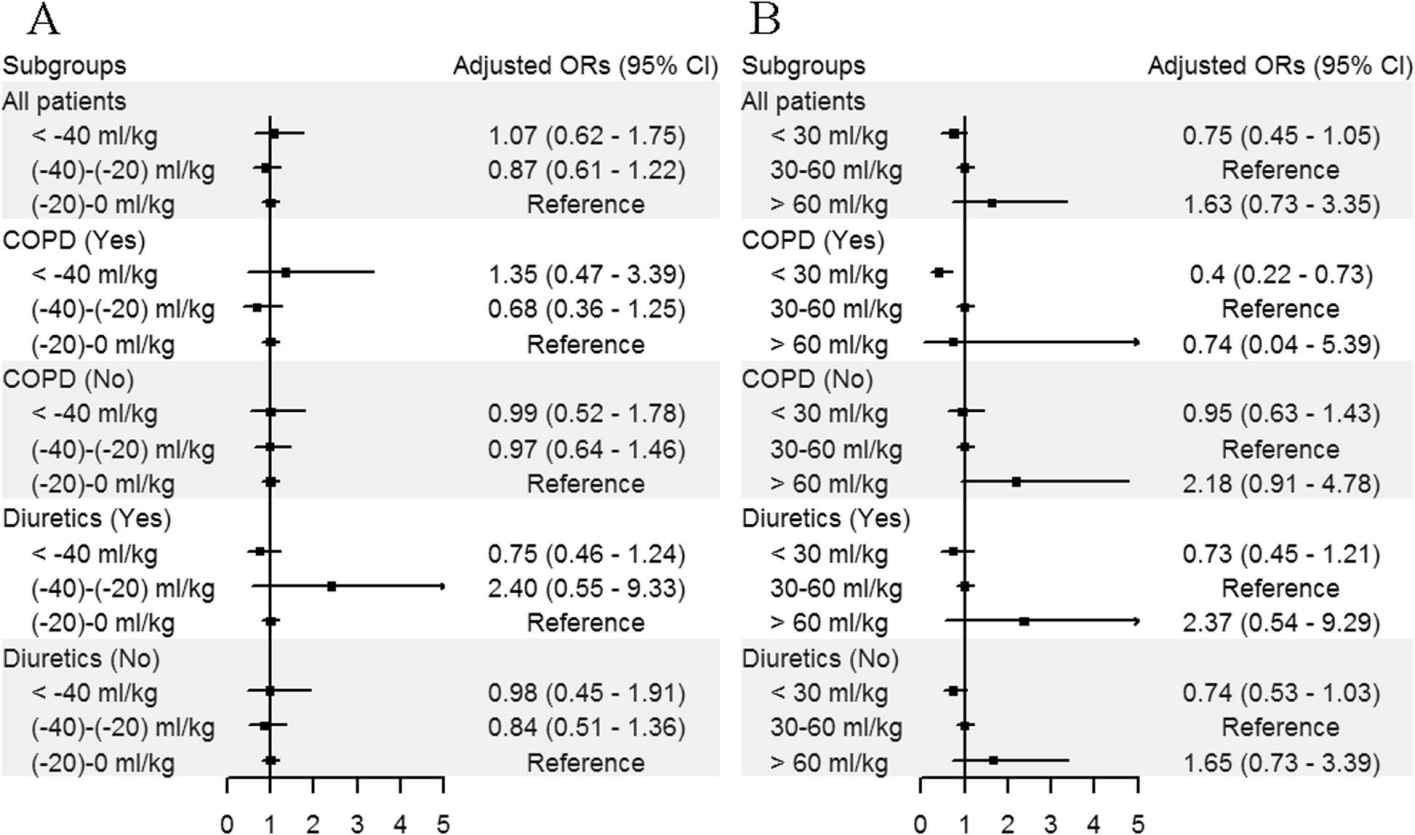
Subgroup analyses using negative fluid balance categories (**A**) and fluid intake categories (**B**) displaying the adjusted odds ratio (OR) for extubation failure. Of note, in COPD patients with negative fluid balance before extubation, lower fluid intake was associated with extubation success. COPD chronic obstructive pulmonary disease

In the subgroup analyses, after adjusting for confounders, FB were not associated with extubation failure in any groups, including patients who received diuretics or had the comorbidity of COPD. However, in the COPD group, patients who received lower level (< 30 mL/kg) fluid intake had higher extubation success than those who received moderate level (30 to 60 ml/kg) fluid intake (OR 0.4, 95% CI [0.22, 0.73], *p* < 0.001, Fig. 2).

## Discussion

In the present study, we found that compared with PFB, NFB was associated with extubation success; However, a more NFB was not associated with improved extubation outcome. Fluid intake within 24 hours before extubation was not associated extubation outcome in overall patients; However, for patients with COPD, lower fluid intake was associated with extubation success. Restricted fluid intake may improve extubation outcome for COPD patients with NFB. Duration of mechanical ventilation, comorbidity of COPD, hypercapnia, use of diuretics, and SAPSIIscore were associated with extubation failure. Fluid intake was associated duration of mechanical ventilation, ICU and hospital length of stay.

The effect of NFB on mortality has been described in critically ill patients [7, 8, 18, 19]. As for extubation outcome, PFB has been suggested as a risk factor for extubation failure. Upadya et al. found patients with NFB were more likely to be successfully weaned than those with PFB [10]. In the study of Vivar et al., PFB 24 hours prior to extubation is a good predictor of extubation failure [11]. A prospective study showed patients who had undergone the weaning protocol had higher probability of weaning success than those who had undergone weaning based in clinical practice. The first step of the institutional weaning protocol is avoidance of PFB for 24 hours prior to the weaning trial [20]. In the present study, PFB had higher percentage of extubation failure, which was in line with these studies. However, the current evidence mainly focused on comparison between PFB and NFB, and whether a more NFB could further increase extubation success NFB remains unclear.

The results of our study showed there was no significant association between the degree of NFB and extubation failure. That may be a complicated question. NFB is associated with extubation success; On the other hand, more NFB is not associated increased probability of extubation success. The potential mechanisms remain unclear. We speculated that the result of achieving NFB may be a surrogate of the recovery of cardiac and renal functions. With the stabilization of the hemodynamic conditions, the degree of NFB mainly depends on the previous fluid accumulation. However, neither patient fluid balance at admission or volume overload at admission could be retrospectively assessed. Also, the fluid balance during hospital stay prior to the period before extubation was unknown. In Dessap’s study [21], which is a multicenter randomized controlled trial, the B-type natriuretic peptide (BNP)-guided fluid management strategy had more negative cumulative and average daily fluid balance during weaning and increased the number of ventilator-free days. However, the BNP-guided group and the usual care group had similar fluid balance on the extubation day or the day after extubation, which suggested there may be less previous fluid accumulation. In addition, BNP-guided group and the usual care group had similar fluid intake during weaning, which suggested fluid intake may have little impact on weaning or extubation outcome.

Administration of diuretics in critically ill patients remains controversial. In the present study, diuretics was associated with extubation failure. Upadya et al. reported that administration of diuretics was associated with NFB, it was not associated with weaning outcomes [10]. A meta-analysis confirmed a conservative or deresuscitative strategy (active removal of fluid using diuretics or renal replacement therapy) resulted in increased ventilator-free days [14]. However, one retrospective cohort study reported that diuretic use was associated with increased mortality in patient with negative fluid balance [22]. Diuretics was significantly associated urine output and fluid balance; However, if the relationship between diuretics and extubation outcome is mediated by fluid balance, the effect of diuretics on extubation outcome could be obscure; Since the relationship between fluid balance and extubation outcome remains uncertain. Considering the substantial potential risks of diuretic administration, the administration of diuretics within 24 hours before extubation for NFB patients should be cautious.

In COPD patients with NFB, the present study showed compared with moderate fluid intake (30 to 60 ml/kg), lower fluid intake (0 to 30 ml/kg) within 24 hours before extubation was associated with extubation success. A prospective observational study found a significant association between PFB in the 48 hours before spontaneous breathing trial (SBT) and SBT failure, but not for the overall patients [23]. In patients with COPD, the exaggerated inspiratory fall of intrathoracic pressure and the increase of myocardial oxygen consumption may make these patients high risk of weaning-induced pulmonary edema and weaning failure [24–26].

The main advantage of the present study is the large sample size. The large size of the cohort allowed to adjust for several confounders. However, there are several limitations to the study. First, the study is a retrospective observational study, when considering the findings, its post hoc nature should be taken into account. Despite the confounders adjusted, several residual potential confounders may also influence extubation failure based on the plurality of often-intertwined mechanisms, such as the respiratory, circulatory, and neurological functions [27]. Second, the generalizability of the study may be in question because it was conducted at a single tertiary care hospital. In addition, there were no available data regarding the weaning protocol, although it is an institutional standard. Third, the net fluid balance and assessment of patients between ICU admission and 24 hours prior to extubation was not included. This period could be days to weeks and would likely have an impact on success or failure than simply looking at 24 hours before extubation, which may merit further research. Fourth, patients who underwent CRRT were excluded, the findings could not be generalized to these patients. Fifth, although we included admitting ICU types, which could not replace the admitting diagnoses. In addition, the reasons for reintubation were not available, which could also cause selected bias.

## Conclusions

In patients with negative fluid balance, a more negative fluid balance or restricted fluid intake were not associated with extubation success. However, in COPD patients, restricted fluid intake was associated with extubation success.

## Supplementary Information

**Additional file 1.**

## Data Availability

The data in the present study is available in the MIMIC-III database (mimic.mit.edu). Researchers can access to the database after the approval of PhysioNet.
