# Supplementary material for: Association between fluid intake and extubation failure in intensive care unit patients with negative fluid balance: a retrospective observational study
Source: BMC Anesthesiol. 2022 Jun 1;22:170. doi: 10.1186/s12871-022-01708-3 (PMC9158150; doi:10.1186/s12871-022-01708-3)
Supplement: Supplementary file 1 — Additional file 1. [file 12871_2022_1708_MOESM1_ESM.docx]

| Variables | Total  (n = 3433) | Negative FB  (n = 1803) | Positive FB  (n = 1630) | P value |
| --- | --- | --- | --- | --- |
| Age, years (median, [IQR]) | 66 (52, 77) | 66 (53, 77) | 65 (51, 78) | 0.317 |
| Sex: male (n (%)) | 1925 (56) | 988 (55) | 937 (57) | 0.121 |
| Weight, Kg (median, [IQR]) | 79 (66, 93) | 80 (67, 95) | 78 (66, 92) | 0.028 |
| Ethnicity, n (%) |  |  |  | 0.664 |
| White | 2465 (72) | 1310 (73) | 1155 (71) |  |
| Asian | 72 (2) | 34 (2) | 38 (2) |  |
| Black | 283 (8) | 146 (8) | 137 (8) |  |
| Hispanic/latino | 99 (3) | 54 (3) | 45 (3) |  |
| Other | 514 (15) | 259 (14) | 255 (16) |  |
| ICU types, n (%) |  |  |  | <0.001 |
| CCU | 413 (12) | 250 (14) | 163 (10) |  |
| CSRU | 512 (15) | 330 (18) | 182 (11) |  |
| MICU | 1319 (38) | 693 (38) | 626 (38) |  |
| SICU | 590 (17) | 265 (15) | 325 (20) |  |
| TSICU | 599 (17) | 265 (15) | 334 (20) |  |
| Comorbidities, n (%) |  |  |  |  |
| Diabetes Mellitus | 889 (26) | 494 (27) | 395 (24) | 0.038 |
| Chronic Heart failure | 1171 (34) | 699 (39) | 472 (29) | <0.001 |
| Hypertension | 1705 (50) | 920 (51) | 785 (48) | 0.1 |
| COPD | 803 (23) | 453 (25) | 350 (21) | 0.013 |
| Liver disease | 382 (11) | 165 (9) | 217 (13) | <0.001 |
| Tumor | 317 (9) | 141 (8) | 176 (11) | 0.003 |
| Renal insufficiency | 526 (15) | 268 (15) | 258 (16) | 0.462 |
| Disease severity score before extubation (median, [IQR]) |  |  |  |  |
| SOFA | 5 (3, 8) | 6 (4, 8) | 5 (3, 8) | <0.001 |
| SAPS II | 41 (32, 51) | 42 (33, 51) | 40 (32, 51) | 0.091 |
| Extubation failure, n (%) | 424 (12) | 201 (11) | 223 (14) | 0.028 |
| Fluid balance (ml/kg/24 hours) | -2 (-16, 13) | -15 (-27, -8) | 14 (6, 25) | <0.001 |
| Total Input (ml/kg/24 hours) | 30 (20, 45) | 24 (15, 34) | 40 (27, 55) | <0.001 |
| Total Output (ml/kg/24 hours) | 32 (20, 47) | 41 (30, 56) | 22 (14, 32) | <0.001 |
| Diuretics, n (%) | 1165 (34) | 697 (39) | 468 (29) | <0.001 |
| Blood products transfusion, n (%) | 790 (23) | 441 (24) | 349 (21) | 0.038 |
| MV duration before extubation, hours | 69 (41, 132) | 87 (51, 147) | 56 (36, 107) | <0.001 |
| Hospital LOS, days (median, [IQR]) | 14 (9, 23) | 15 (9, 24) | 14 (8, 23) | <0.001 |
| ICU LOS, days (median, [IQR]) | 7 (4, 13) | 8 (4, 13) | 6 (3, 12) | <0.001 |
| Hospital mortality, n (%) | 475 (14) | 213 (12) | 262 (16) | <0.001 |

S-Table 1 Comparison of patient characteristics between positive and negative fluid balance

IQR interquartile range, ICU intensive care unit, CCU Coronary Care Unit, CSRU Cardiac Surgery Recovery Unit, TSICU Trauma Surgical ICU, MICU Medical ICU, SICU Surgical ICU, COPD chronic obstructive pulmonary disease, SOFA sequential organ failure assessment score, SAPS II simplified acute physiology score, LOS length of stay.
